# Validity of a wireless instrumented insole (WalkinSense system) for measuring gait metrics

**DOI:** 10.1002/jeo2.70438

**Published:** 2025-09-27

**Authors:** Melanie Eckelt, Jennifer Fayad, Anne Backes, Gaëlle Schurmans, Frederic Garcia, Bernd Grimm, Valeria Serchi, Tobias Meyer, Thomas Solignac, Caroline Mouton, Romain Seil, Laurent Malisoux

**Affiliations:** ^1^ Department of Precision Health Luxembourg Institute of Health Strassen Luxembourg; ^2^ Centre Hospitalier de Luxembourg Luxembourg City Luxembourg; ^3^ IEE S.A. Bissen Luxembourg

**Keywords:** gait analysis, instrumented insoles, rehabilitation, validation, wearables

## Abstract

**Purpose:**

Gait analysis has become a valuable tool in assessing abnormal gait patterns and quantifying improvements resulting from interventions, particularly in the rehabilitation of orthopaedic patients. Wearables can measure gait metrics in daily life settings, but they must first be validated before being applied in such contexts. This study aims to assess the validity of a wireless instrumented insole (WalkinSense).

**Methods:**

Recordings of 104 healthy participants were obtained at various speed and slope conditions (3 km/h, 4.5 km/h [−3°, −6°, +3° and +6°], 6 km/h and 9 km/h). Spatiotemporal and kinematic variables were collected simultaneously with an instrumented treadmill, a three‐dimensional motion capture system and with the WalkinSense system. Mean bias between the systems was assessed using separate Bland–Altman analyses for each metric and condition. Mean error and limits of agreement (absolute and percentage) were calculated and the agreement was statistically quantified using a priori set thresholds (excellent <5%, good <10%, acceptable <15% and poor >15%). MAPE scores and a two‐way mixed model intraclass correlation coefficient (ICC) for consistency were also calculated.

**Results:**

All spatiotemporal variables (except double support time) showed good or excellent agreement, MAPE scores lower than 5% and ICC values > 0.88 in the walking speeds. Data collected with the WalkinSense system showed acceptable or good agreement for the spatiotemporal variables in running. Kinematic variables showed only poor agreements across all speeds and slopes.

**Conclusion:**

These findings suggest that the WalkinSense system may be useful to quantify spatiotemporal variables with good to excellent accuracy across various walking speeds. However, based on the results of this study indicate that the WalkinSense system is not suitable for measuring kinematic variables without substantial improvements.

**Level of Evidence:**

Level II, diagnostic studies.

## Graphical abstract

This study assessed the validity of the WalkinSense system (WSS) as a portable alternative to instrumented force plates for measuring spatiotemporal and kinematic gait variables. Data from 101 participants across different speeds and slopes showed accurate agreement for most spatiotemporal variables. Kinematic and running data need refinement. WSS appears suitable for real‐life gait assessment outside laboratory settings.

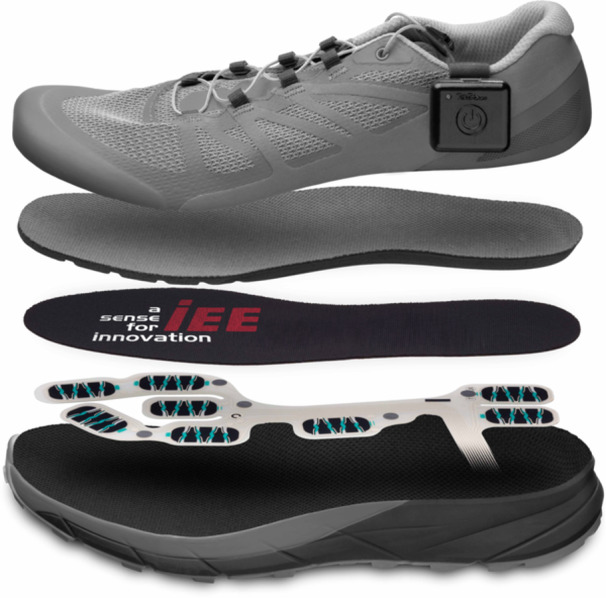

**Abbreviations**
GRF: ground reaction force
HD‐FSR: high dynamic force sensing resistor
IC: initial contact
ICC: intraclass correlation coefficient
IMU: inertial measurement units
IQR: interquartile range
MAPE: mean absolute percentage error
SD: standard deviation
TO: toe‐off
WSS: WalkinSense system

## INTRODUCTION

Gait analysis is the standard approach used to quantify human locomotion [29]. It has become a well‐established tool in sports science and biomechanical research, but is now also a very valuable instrument in clinical diagnostics, monitoring functional recovery and musculoskeletal rehabilitation. The benefits of gait analysis to quantify gait disturbances have become apparent in different medical areas, especially in orthopaedics. In fact, it proved to help better understand, objectify and quantify the individual gait and movement disorders to optimise patient‐specific therapy [14]. Motion capture systems, force plates and instrumented treadmills are currently considered as the gold standards for quantitative gait analysis [28]. However, such equipment is expensive, resource‐intensive, and limited to stationary use in laboratory environments. In fact, its application is primarily confined to research centres and, due to its stationary nature, only provides momentary views of a patient's gait [2].

In rehabilitation, clinicians are constantly aiming to assess the improvements in patients following interventions [20]. The idea that instrumented insoles could help monitor patients and enable gait analysis to be transferred from the laboratory to everyday life has been around for a long time [4, 18, 22]. Assessing gait in daily life provides valuable information for clinicians, as it captures real‐life conditions, including environmental influences and natural dual‐tasking scenarios [5].

A number of wearable gait assessment systems have been developed for use in clinical and home‐care settings. However, many of these systems face limitations that restrict their applicability in routine clinical practice, including orthopaedics and sports medicine. For example, systems relying solely on inertial measurement units (IMUs) often lack the ability to capture spatial gait parameters or detailed gait events such as initial contact and toe‐off, which can be more precisely identified using pressure‐sensitive insoles [24]. Furthermore, some systems are not designed for long‐term monitoring or unsupervised home use, limiting their potential for continuous gait tracking outside laboratory settings. While Roth et al. [24] demonstrated the feasibility of synchronised pressure‐sensitive insoles for home monitoring, their work focused on a research prototype rather than a commercially available product. This highlights the ongoing need for validated, user‐friendly, and clinically applicable solutions that combine pressure and inertial data for comprehensive gait assessment in real‐world environments. The new wireless instrumented insole system (WalkinSense system; WSS; IEE S.A.) consists of pressure‐sensitive insoles combined with inertial measurement units (IMU). It uses printed force‐sensitive resistor technology that results in a uniquely thin and soft insole not altering the height, stiffness or feeling of the shoe, maintaining natural gait and original shoe comfort.

However, gait analysis must meet sufficient scientific quality standards [1]. Although some wearable gait analysis devices, such as the ARION insoles [27], have demonstrated good validity for spatiotemporal parameters, these findings are often based on relatively small sample sizes and limited testing conditions. Many commercially available wearables have yet to undergo comprehensive scientific validation across diverse populations, walking speeds, slopes, and gait parameters. Therefore, the main objectives of this study were to assess the validity and accuracy of the WSS by comparing the gait metrics calculated by the device against gold standard laboratory equipment under a broad range of conditions. The analysis focused on spatiotemporal and kinematic variables, as these are commonly used as reference values for detecting gait abnormalities [9, 13].

## METHODS

### Participants

In total, 104 healthy participants without history of surgery of the lower limbs or orthopaedic conditions (50% females, age 35.6 ± 11.7 years, body height 172 ± 10 cm; body mass 70.7 ± 14.4 kg) volunteered to participate in this study. The number of participants required for assessing agreement between measurement methods varied from 39 to 83 across the main metrics, according to the approach by Lu et al. [17]. To account for potential data loss caused by technical issues, our goal was to collect data from 100 healthy participants. The study was approved by the relevant national ethics committee (approval ref: 202212/04 Version 2.0). Participants were provided with a detailed explanation of the study protocol and gave their informed consent prior to participation.

Information on age, height and body mass was collected and each participant was provided with WSS insoles matching their shoe size. The participants wore their own familiar sports shoes for data collection. Reflective markers were placed on the shoes, two on the heel (back and lateral) and three on the metatarsals (1, 2 and 5) for the collection of kinematic data.

### Instruments

**Figure 1:**
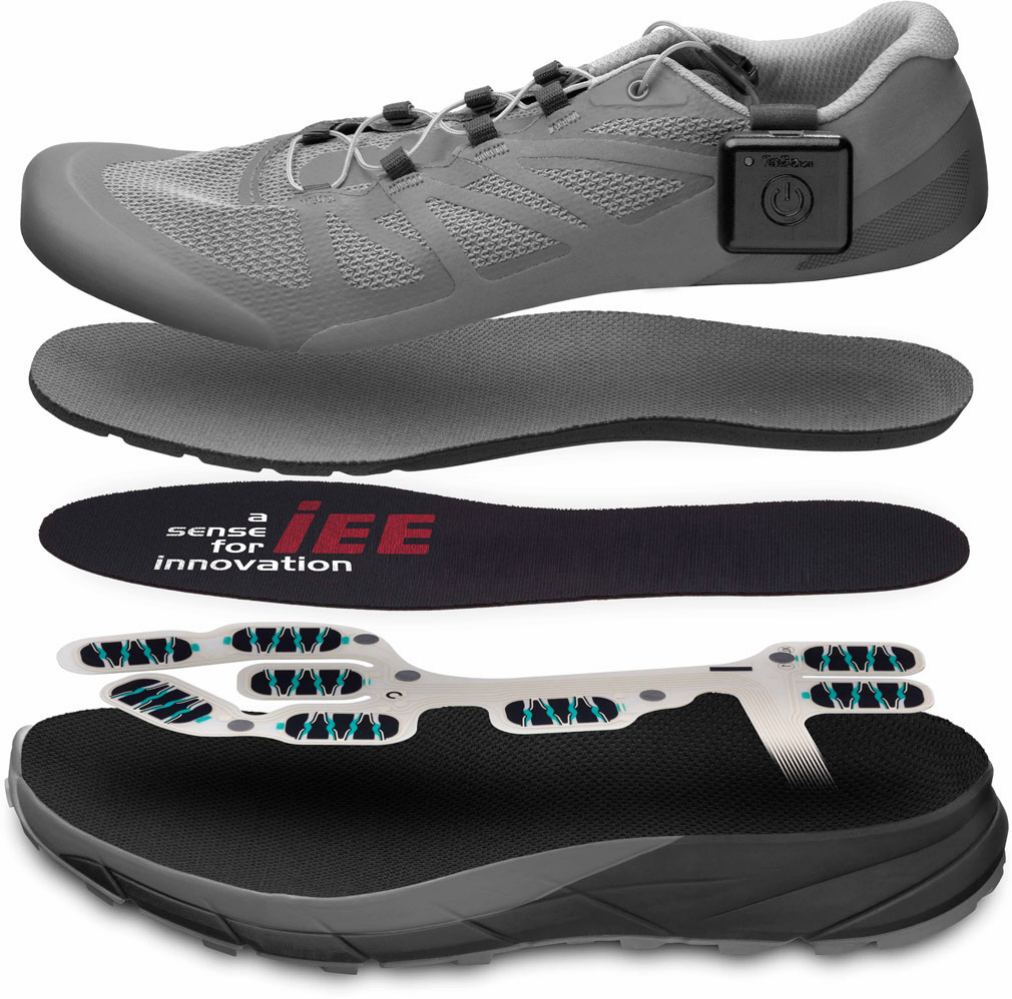
The WalkinSense system (IEE S.A.).

The WSS (Figure 1) consists of a hardware environment with both IMU (ICM‐20948) and insoles for data acquisition. The pressure‐sensitive insoles are equipped with eight High Dynamic Force Sensing Resistor (HD‐FSR cells) sensors, whose location and size have been established as a reliable standard for plantar pressure sensor positioning [21]. The WSS hardware is small and light and thus provides a low potential interference with gait or optical appearance. With a total height of 0.350 mm, the insoles can be placed into many types of footwear and can be used with the patient's own shoes and insoles. The WSS combines a long battery life (up to 10 days) with a sampling frequency up to 200 Hz.

All trials were performed on a split‐belt treadmill instrumented with force plates (M‐Gait, Motek Forcelink Amsterdam). The three‐dimensional ground reaction forces (GRF) were recorded at 1 kHz. Kinematic data was collected using a three‐dimensional motion capture system with eight infra‐red‐cameras (300 Hz, Migus, Qualisys AB, Gothenburg, Sweden).

### Data collection

Prior to data collection, the participants were instructed to walk on the treadmill at 3 km/h with no slope to familiarise themselves with the experimental settings. The participants completed a series of nine trials. Each of the nine trials consisted of a one‐minute habituation phase followed by a one‐minute data recording. The trials were always conducted in the same predetermined order: walking at slow speed (3 km/h) with no slope, walking at middle speed (4.5 km/h) with no slope, with positive slope of 3° and 6° and with negative slopes of −3° and −6°, walking at high speed (6 km/h) with no slope and running (9 km/h) with no slope. Data collection from the WSS was synchronised with multiple systems using a master‐slave setup with hardware triggering. Thus, data collection from the WSS (pressure and IMU data), the treadmill, and the motion capture systems were conducted simultaneously through the Qualisys Sync Box.

### Data processing

The WSS was setup to record data at a sampling frequency of 100 Hz. The IMU's gyroscope and accelerometer were set up with a full‐scale range of ±1000 dps and ±8 G, respectively. The recorded data from the WSS, the treadmill and the motion capture system signals were resampled to 200 Hz, enabling data analysis. The force data was filtered with a bidirectional second order low‐pass Butterworth filter with a cut‐off frequency of 10 Hz for the walking conditions and 30 Hz for the running condition to minimise the noise coming from the treadmill. Initial contact (IC) and toe‐off (TO) were identified by the vertical GRF exceeding or falling below a 20 N threshold, respectively [8]. Marker data were tracked and processed using Qualisys Track Manager (Qualisys AB, Gothenburg Sweden) and subsequently exported to Visual3D (HAS‐Motion, Canada) for the calculation of kinematic variables. Kinematic angles were measured as the angle between the line connecting the heel back and second metatarsal and the ground.

The WSS system features an embedded library (Walkinsense Analytics Library v1.0.00) for the implementation of the spatiotemporal and kinematic variables, which are described in Table 1. The manufacturer provides a step detector based on the pressure activation profile derived from the insoles and combines the information from the IMU and the insoles to derive the position and orientation of the foot over time. Relevant metrics about the gait cycle timing and 3D change over time are then computed for each of the detected steps. The WSS provides as output the step‐by‐step metrics and temporal gait events timing.

**Table 1:** Gait metrics considered in this study.

	Variable	Description	WSS Sensors
Spatiotemporal variables	Stance time (s)	Time between IC and TO	Pressure‐sensitive insoles
	Swing time (s)	Time between TO and IC	Pressure‐sensitive insoles
	Stride time (s)	Time between 2 subsequent IC of the same foot	Pressure‐sensitive insoles
	Stride length (m)	Product of speed (treadmill) and stride time	Pressure‐sensitive insoles + IMU
	Single support time (s)	Time between TO of the opposite leg to IC of the opposite leg	Pressure‐sensitive insoles
	Double support time (s)	Stance time minus single support time	Pressure‐sensitive insoles
Kinematic variables	Foot‐ground angle at IC (°)	Heel‐toe plane angle to horizontal at IC	Pressure‐sensitive insoles + IMU
	Foot‐ground angle at TO (°)	Heel‐toe plane angle to horizontal at TO	Pressure‐sensitive insoles + IMU

Abbreviations: IC, initial contact; IMU, inertial measurement unit; TO, toe‐off.

A dedicated Python algorithm (Spyder V.5.4.3; Python V.3.12) to select the corresponding steps detected for both the WalkinSense and ground truth systems. This allowed for the exclusion of the any erroneous steps (e.g., crossing the treadmill midline) from the 60‐s recordings and to make the comparison of the systems based on the same set of steps.

Gait metrics were calculated for each gait cycle, and then averaged per participant and left/right limbs.

### Statistical analysis

All statistical analyses were conducted using R with RStudio (V.4.3.2). Mean and standard deviation (SD) were calculated independently for every gait metric measured at each condition. Mean bias between the WSS and the gold standard was assessed using separate Bland–Altman analyses for every gait metric and condition in the original units. In the Bland–Altman analysis, the differences between two instruments are plotted against their mean value. This leads to a visualisation of the agreement between them [6]. Mean error and limits of agreement (absolute and percentage) were calculated and used to objectively assess the agreement between the systems using a statistical approach recommended by Shieh [26]. This method assesses the mean difference and variability of the difference between the systems with respect to a pre‐defined threshold, whereby a certain proportion of the data should lie within the threshold in order to declare agreement. Agreement was quantified using pre‐established thresholds (excellent <5%, good <10%, acceptable <15% and poor >15%). These thresholds are in accordance with the proposed accuracy thresholds for consumer and clinical wearables [11, 27].

An absolute error measure was used to enable easier comparison between instruments across all test conditions and variables investigated [11]. The mean absolute percentage error (MAPE), a commonly used measure of forecast accuracy [12], was calculated as the mean of the absolute differences between the WSS system and the ground truth, divided by the ground truth value and multiplied by 100. The following ranges were proposed for the interpretation of the MAPE score: less than 10% highly accurate, 11%–20% good, 21%–50% reasonable and more than 51% inaccurate forecast [16].

A two‐way mixed model intraclass correlation coefficient (ICC 3,1) for consistency was calculated using the “irr” package. ICCs were interpreted as poor (<0.50), moderate (0.50 < 0.75), good (0.75 < 0.90) or excellent (≥0.90) [15].

## RESULTS

From the 104 participants tested, three participants were excluded from the analysis because of a too noisy signal from the WSS (*n* = 1) and data were considered as extreme outliers according to the interquartile range (IQR) method (*n* = 2). Furthermore, due to some missing data (e.g., participants could not run at 9 km/h), some test conditions (speeds and slopes) may include fewer participants (see Table 2).

**Table 2:** Descriptive statistics, percentage differences with 95% limits of agreement and corresponding thresholds, MAPE score and ICC values for all test conditions (speed and slope).

Condition (km/h; °)	*n*	Ground truth (mean ± SD)	WSS (mean ± SD)	Mean ± SD percentage differences [95% limits of agreement]	MAPE (%)	ICC (3,1)
Stance time (s)						
3; 0	98	0.825 ± 0.063	0.832 ± 0.066	0.742 ± 1.825 [−2.835 to 4.318]***	1.5	0.97
4.5; 0	101	0.682 ± 0.039	0.692 ± 0.043	1.329 ± 1.993 [−2.577 to 5.236]***	1.8	0.94
4.5; −3	100	0.672 ± 0.039	0.680 ± 0.044	1.134 ± 2.292 [−3.358 to 5.625]***	1.8	0.93
4.5; −6	100	0.656 ± 0.041	0.663 ± 0.046	0.988 ± 2.156 [−3.238 to 5.213]***	1.7	0.94
4.5; 3	100	0.694 ± 0.045	0.705 ± 0.049	1.517 ± 2.141 [−2.679 to 5.714]***	2.0	0.94
4.5; 6	100	0.699 ± 0.045	0.712 ± 0.049	1.760 ± 2.536 [−3.211 to 6.731]***	2.4	0.92
6; 0	100	0.600 ± 0.033	0.610 ± 0.038	1.515 ± 2.825 [−4.023 to 7.053]**	2.2	0.88
9; 0	98	0.293 ± 0.028	0.338 ± 0.036	12.832 ± 5.090 [2.856 to 22.807]**	15.2	0.80
Swing time (s)						
3; 0	98	0.442 ± 0.037	0.435 ± 0.038	−1.706 ± 3.710 [−8.977 to 5.564]**	2.9	0.92
4.5; 0	101	0.396 ± 0.024	0.385 ± 0.026	−2.801 ± 3.999 [−10.638 to 5.037]**	3.2	0.84
4.5; −3	100	0.395 ± 0.025	0.386 ± 0.030	−2.436 ± 4.272 [−10.809 to 5.936]**	3.0	0.84
4.5; −6	100	0.391 ± 0.027	0.384 ± 0.031	−2.090 ± 3.941 [−9.814 to 5.634]**	2.9	0.87
4.5; 3	100	0.401 ± 0.027	0.389 ± 0.030	−3.146 ± 4.468 [−11.904 to 5.611]**	3.6	0.85
4.5; 6	100	0.402 ± 0.029	0.389 ± 0.034	−3.743 ± 5.047 [−13.634 to 6.148]**	4. 2	0.84
6; 0	100	0.373 ± 0.022	0.363 ± 0.028	−3.083 ± 5.140 [−13.158 to 6.991]*	3.6	0.77
9; 0	98	0.461 ± 0.041	0.411 ± 0.039	−12.573 ± 10.280 [−32.722 to 7.577]*	10.6	0.61
Stride time (s)						
3; 0	98	1.267 ± 0.094	1.266 ± 0.093	−0.038 ± 0.061 [−0.158 to 0.083]***	0.1	1.00
4.5; 0	101	1.078 ± 0.057	1.077 ± 0.057	−0.068 ± 0.170 [−0.402 to 0.266]***	0.1	0.99
4.5; −3	100	1.067 ± 0.061	1.066 ± 0.061	−0.049 ± 0.072 [−0.190 to 0.091]***	0.1	1.00
4.5; −6	100	1.047 ± 0.065	1.046 ± 0.065	−0.042 ± 0.049 [−0.138 to 0.055]***	0.1	1.00
4.5; 3	100	1.095 ± 0.067	1.095 ± 0.067	−0.044 ± 0.058 [−0.159 to 0.071]***	0.1	1.00
4.5; 6	100	1.101 ± 0.069	1.101 ± 0.069	−0.064 ± 0.167 [−0.391 to 0.264]***	0.1	1.00
6; 0	100	0.973 ± 0.051	0.972 ± 0.051	−0.048 ± 0.054 [−0.155 to 0.058]***	0.1	1.00
9; 0	98	0.755 ± 0.054	0.749 ± 0.045	−0.731 ± 3.517 [−7.624 to 6.163]**	0.6	0.85
Stride length (m)						
3; 0	98	1.056 ± 0.078	1.062 ± 0.081	0.552 ± 1.448 [−2.286 to 3.390]***	1.2	0.98
4.5; 0	101	1.347 ± 0.072	1.344 ± 0.073	−0.290 ± 1.370 [−2.976 to 2.395]***	0.9	0.97
4.5; −3	100	1.334 ± 0.076	1.326 ± 0.082	−0.635 ± 1.493 [−3.560 to 2.291]***	1.2	0.97
4.5; −6	100	1.309 ± 0.081	1.290 ± 0.086	−1.506 ± 1.591 [−4.624 to 1.613]***	1.7	0.97
4.5; 3	100	1.369 ± 0.084	1.370 ± 0.085	0.048 ± 1.207 [−2.318 to 2.413]***	0.9	0.98
4.5; 6	100	1.377 ± 0.087	1.374 ± 0.089	−0.233 ± 1.158 [−2.502 to 2.036]***	0.9	0.98
6; 0	100	1.621 ± 0.086	1.608 ± 0.089	−0.868 ± 1.452 [−3.714 to 1.978]***	1.2	0.97
9; 0	98	1.886 ± 0.136	1.806 ± 0.129	−4.551 ± 4.573 [−13.515 to 4.413]**	4.2	0.81
Single support time (s)						
3; 0	98	0.442 ± 0.037	0.435 ± 0.038	−1.727 ± 3.713 [−9.004 to 5.550]**	2.9	0.92
4.5; 0	101	0.396 ± 0.024	0.385 ± 0.026	−2.855 ± 3.997 [−10.689 to 4.980]**	3.2	0.84
4.5; −3	100	0.395 ± 0.025	0.386 ± 0.030	−2.544 ± 4.251 [−10.877 to 5.788]**	3.1	0.84
4.5; −6	100	0.391 ± 0.027	0.383 ± 0.031	−2.160 ± 3.907 [−9.817 to 5.498]**	2.9	0.88
4.5; 3	100	0.401 ± 0.027	0.389 ± 0.030	−3.215 ± 4.467 [−11.970 to 5.540]**	3.6	0.85
4.5; 6	100	0.402 ± 0.029	0.389 ± 0.034	−3.798 ± 5.040 [−13.677 to 6.081]**	4.2	0.84
6; 0	100	0.373 ± 0.022	0.362 ± 0.026	−3.254 ± 4.888 [−12.833 to 6.326]**	3.5	0.80
Double support time (s)						
3; 0	98	0.383 ± 0.044	0.397 ± 0.053	2.914 ± 7.307 [−11.408 to 17.235]*	6.7	0.81
4.5; 0	101	0.286 ± 0.028	0.306 ± 0.043	5.787 ± 8.048 [−9.987 to 21.560]	8.7	0.69
4.5; −3	100	0.278 ± 0.026	0.295 ± 0.045	4.419 ± 13.473 [−21.987 to 30.825]	8.6	0.62
4.5; −6	100	0.265 ± 0.026	0.279 ± 0.044	3.977 ± 11.197 [−17.970 to 25.924]	8.5	0.68
4.5; 3	100	0.293 ± 0.031	0.316 ± 0.046	6.483 ± 8.512 [−10.200 to 23.166]	9.7	0.69
4.5; 6	100	0.297 ± 0.030	0.323 ± 0.048	7.164 ± 10.880 [−14.160 to 28.489]	11.3	0.59
6; 0	100	0.227 ± 0.021	0.248 ± 0.041	6.704 ± 13.457 [−19.671 to 33.079]	11.5	0.52
Foot ground angle at initial contact (°)						
3; 0	98	14.829 ± 4.412	16.913 ± 3.871	11.168 ± 22.057 [−32.064 to 54.400]	26.7	0.63
4.5; 0	101	20.324 ± 4.251	22.647 ± 3.871	9.161 ± 17.544 [−25.224 to 43.547]	20.4	0.47
4.5; −3	100	22.140 ± 4.727	25.088 ± 4.206	10.762 ± 17.727 [−23.983 to 45.508]	21.5	0.48
4.5; −6	100	23.644 ± 4.442	27.527 ± 4.364	13.354 ± 14.473 [−15.013 to 41.722]	21.1	0.56
4.5; 3	100	17.668 ± 4.358	19.071 ± 4.000	6.096 ± 19.709 [−32.534 to 44.726]	20.1	0.60
4.5; 6	100	14.518 ± 4.243	15.652 ± 4.102	5.215 ± 22.606 [−39.092 to 49.522]	22.9	0.65
6; 0	100	26.263 ± 4.168	28.100 ± 4.064	5.507 ± 15.096 [−24.081 to 35.095]	15.2	0.43
9; 0	98	11.542 ± 4.270	20.434 ± 6.559	72.273 ± 261.425 [−440.121 to 584.666]	97.1	0.56
Foot ground angle at toe off (°)						
3; 0	98	50.344 ± 5.344	49.122 ± 5.521	−2.921 ± 9.125 [−20.805 to 14.964]	7.1	0.69
4.5; 0	101	60.474 ± 4.939	59.733 ± 6.477	−1.631 ± 8.043 [−17.395 to 14.134]	6.3	0.60
4.5; −3	100	58.241 ± 5.136	56.583 ± 7.308	−3.494 ± 10.353 [−23.786 to 16.798]	6.9	0.54
4.5; −6	100	56.042 ± 5.495	52.974 ± 9.043	−6.643 ± 11.410 [−29.006 to 15.721]	8.7	0.60
4.5; 3	100	62.018 ± 4.787	61.739 ± 6.412	−0.898 ± 8.311 [−17.188 to 15.391]	6.4	0.57
4.5; 6	100	63.929 ± 5.007	63.416 ± 6.732	−1.321 ± 9.221 [−19.395 to 16.753]	6.5	0.50
6; 0	100	68.271 ± 4.975	67.472 ± 6.279	−1.904 ± 10.315 [−22.123 to 18.314]	5.8	0.52
9; 0	98	50.398 ± 5.310	53.778 ± 12.387	4.913 ± 12.764 [−20.105 to 29.930]	11.6	0.51

*Note*:***Excellent agreement (<5% error);**Good agreement (<10% error);*Acceptable agreement (<15% error). No star indicates poor agreement (≥15% error).

Abbreviations: ICC, intraclass correlation coefficient; MAPE, mean absolute percentage error; SD, standard deviation; WSS, WalkinSense system.

Table 2 presents the mean ± SD values from the WSS and the Motek/Qualisys system, considered as the ground truth, the mean ± SD values of the percentage differences with 95% limits of agreement and the corresponding thresholds, MAPE scores and ICC values for all gait metrics and test conditions.

The mean percentage difference between WSS and the ground truth data varies between poor and excellent across the gait metrics and test conditions (Table 2). Excellent agreement was achieved for stride time and stride length across all conditions except running, which showed good agreement. For stance time, excellent agreement was achieved for six out of eight test conditions, with the remaining two conditions (6 km/h and 9 km/h) showing good agreement. Good agreement was achieved for swing time at 3 km/h and 4.5 km/h and acceptable agreement at 6 km/h and 9 km/h, whereas single support time showed good agreement across all speed conditions. Poor agreement was found for double support time across all conditions except for 3 km/h, where acceptable agreement was achieved. Kinematic variables showed poor agreement across all speed conditions. Overall, slope did not affect the agreement between the systems.

Overall, the MAPE scores (Table 2) showed highly accurate forecast values across the spatiotemporal variables except for stance time and swing time in running and double support time in walking (6 km/h and 4.5 km/h + 6°), which showed good forecasting values. The MAPE scores for the foot ground angle at TO showed highly accurate forecast values for the seven walking conditions and good forecast values for the running condition. Foot ground angle at IC showed reasonable forecast values for the slow and normal walking conditions, good forecast values for fast walking and a poor forecast for running.

**Figure 2:**
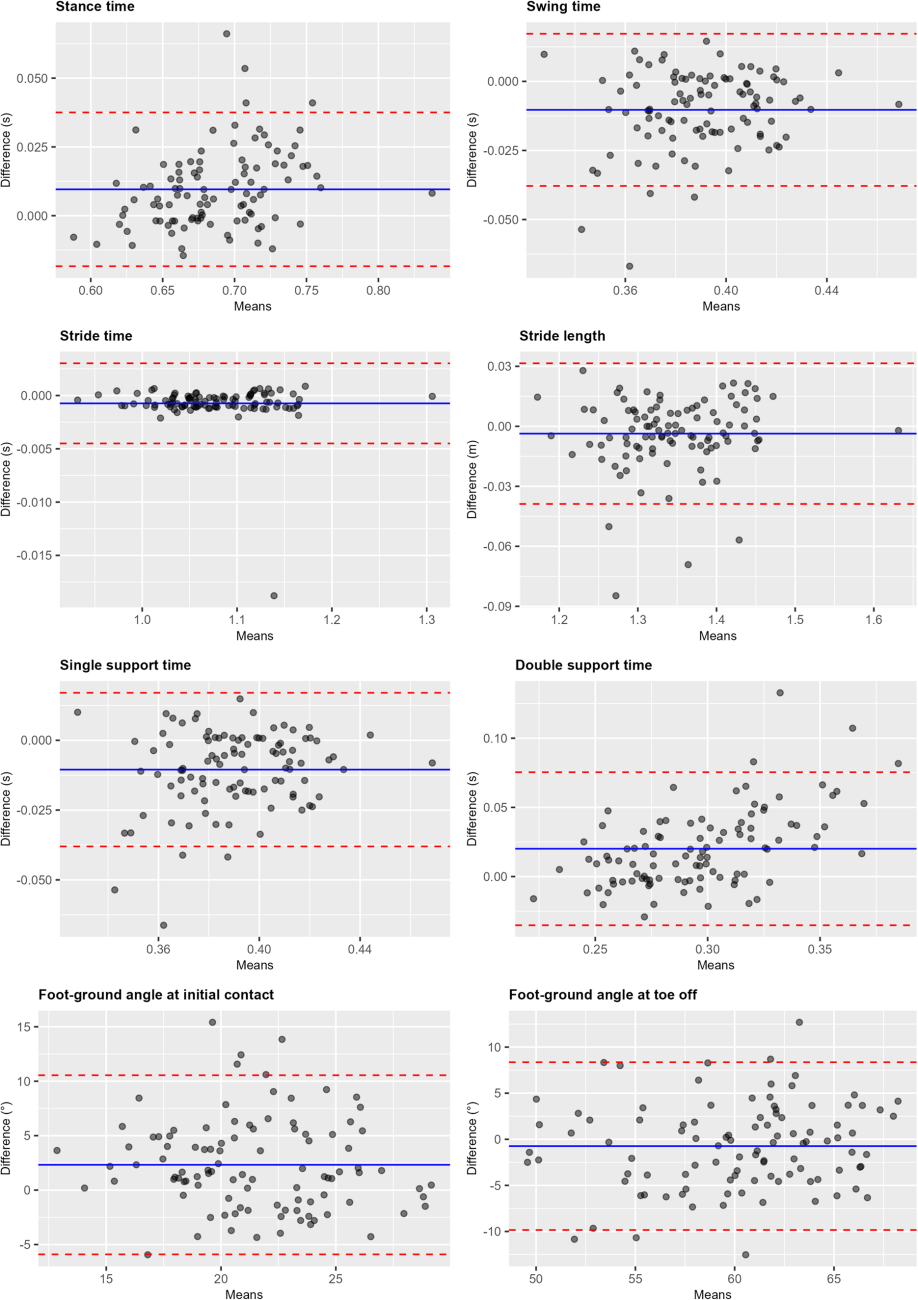
Bland–Altman plots for different gait metrics at 4.5 km/h (no slope).

Figure 2 presents the results of the Bland‐Altman analysis for all gait metrics at 4.5 km/h (no slope). For most parameters, the limits of agreement were narrow and the mean bias was close to zero, indicating good agreement. For example, the mean bias was 0.010 s for stance time (95% limits of agreement: −0.018 to 0.038 s), −0.010 s for swing time (−0.038 to 0.017 s), and −0.001 s for stride time (−0.004 to 0.003 s). Stride length showed a mean bias of −0.004 m (−0.039 to 0.031 m), and single support time −0.011 s (−0.038 to 0.017 s). In contrast, larger mean biases and wider limits of agreement were observed for double support time (0.020 s; −0.035 to 0.075 s) and the two kinematic variables: foot‐ground angle at initial contact (2.323°; −5.902 to 10.548°) and at toe‐off (−0.741°; −9.843 to 8.360°). No systematic overestimation or underestimation was observed. Further Bland‐Altman analyses for all test conditions are provided in the supplementary file. Bland–Altman plots did not indicate systematic patterns of bias for the agreement between the two systems across all conditions and variables (Figure 2 and Supporting Information: Files).

ICC values for the spatiotemporal variables indicated a moderate to excellent consistency across test conditions, with stride time and stride length having the highest ICC and only excellent values for all walking speed conditions and good values in running. ICC values for stance time and swing time indicated excellent consistency at 3 km/h. Stance time also showed excellent values for walking at 4.5 km/h (all slope conditions) and good values at 6 km/h and 9 km/h, whereas ICC values for swing time were good at higher walking speeds (4.5 km/h and 6 km/h) and moderate for running. ICC values for single support time ranged from good (4.5 –9 km/h) to excellent (3 km/h) and for double support time from moderate (4.5–9 km/h) to good (3 km/h), with both having the lowest values at running. ICC values for the kinematic values present poor and moderate consistency (Table 2).

## DISCUSSION

This study aimed to assess the validity and accuracy of the WSS for measuring spatiotemporal and kinematic gait parameters in comparison to gold standard methods. The results demonstrated good agreement for most spatiotemporal parameters across a wide range of walking and running conditions. However, kinematic variables (i.e., foot‐ground angle at initial contact and toe‐off) showed lower agreement. These findings suggest that the WSS is a valid tool for capturing spatiotemporal gait metrics but is currently not suitable for measuring angular kinematics without substantial further improvement.

Compared to earlier studies with sample sizes between 12 and 39 participants [2, 3, 7, 10, 22, 24, 25, 29], this work included a substantially larger and more heterogeneous sample (*N*  = 104) and examined the influence of different walking and running speeds, allowing for a more comprehensive evaluation of system performance under varied conditions. Various positive and negative slopes were also investigated at 4.5 km/h to analyse whether the slope may affect measurement quality at common walking speed. Given the size and heterogeneity of the present sample, the data set is most likely sufficient to reflect the inter‐individual variability of human gait [29]. No data had to be excluded due to hardware malfunctions, except a few file‐saving issues (1.73%). Overall, only three participants out of the 104 initially tested were excluded due to bad quality data, and <2% of the remaining recordings could not be properly analysed, indicating good technical robustness of the WSS under controlled laboratory settings. In contrast, earlier studies using insole‐based systems reported higher exclusion rates due to technical issues. For example Roth et al. [24] had to exclude 13.33% of participants from their study due to technical problems using a custom‐built insole system. These results support the potential of the WSS as a reliable tool for use in research and applied settings, at least with regard to spatiotemporal gait parameters.

Based on the results of the current study, the WSS seems to perform as well as the instrumented treadmill in measuring spatiotemporal walking variables. Mean differences were close to zero in all Bland‐Altman‐Plots, and except for double support time, all spatiotemporal variables showed good to excellent agreement in walking at different speeds and slopes (3–6 km/h; −6° to +6° at 4.5 km/h). In contrast to OpenGo, for example [22], the mean differences are not consistently negative. There are generally higher mean values for stance time and double support time for the WSS, and lower values for swing, stride and single support time, indicating that the WSS does not consistently overestimate or underestimate these metrics. Oerbekke et al. [22] demonstrated only acceptable agreements for the spatiotemporal variables using OpenGo at a single and self‐selected comfortable walking speed (mean ~5.3 km/h), whereas the present study investigated the WSS across multiple speeds (and slopes), and shows good to excellent agreements for the spatiotemporal variables in walking, with the exception of double support time.

The results for double support time were less favourable compared to the other spatiotemporal variables, showing acceptable agreement at 3 km/h but poor agreement for all other test conditions. Interestingly, single support time and swing time are only slightly longer, but still show better agreement. This suggests that the limited sampling frequency (100 Hz) is not the only explanation. Actually, the calculation of double support time requires precise detection of IC and TO for each foot as well as accurate synchronised data [24], which could be a reason for the limited agreement. Compared to walking speeds, running showed slightly weaker results across all metrics. While Van Hooren et al. [27] found excellent agreement for stride time and good agreement for swing time at higher running speeds with Arion, the WSS was able to obtain acceptable to good agreements. However, despite the importance of running assessments in athletic rehabilitation (e.g., following ACL injury), high‐speed running is less frequently assessed in clinical settings focused on pathological gait patterns (e.g., neurological or geriatric populations). Our findings therefore primarily inform the clinical use of the WSS in standard walking and running conditions.

In this study, all spatiotemporal variables (except double support time) showed MAPE scores beneath 5% in the walking test conditions, which is considered a highly accurate prediction. Furthermore, it has been suggested that wearables should have an error of less than 5% for use in clinical trials, while an error of 10%–15% may be acceptable for the general population [11]. This indicates that the WSS is suitable for both settings. The calculated ICC values for the spatiotemporal variables during walking are also comparable with results from other studies, or even slightly better in some cases. For example, Braun et al. [2] were able to reveal an ICC value of 0.84 for stance time with OpenGo, while the WSS showed ICC values > 0.88 for all walking speed. In the present study, both positive and negative slopes at a walking speed of 4.5 km/h did not negatively affect the quality of the measurements, as indicated by the agreement, MAPE scores and ICC values.

The kinematic variables showed only poor agreements, relatively high MAPE scores, and low ICC values, especially at IC. It is important to note that these variables were computed using data from both the insoles and the IMU. Previous studies have raised concerns about the use of IMU systems on shoes, as the movement of the IMU relative to the shoe may lead to less accurate results [10]. Furthermore, it has already been shown that one of the main limitations of the IMU is its tendency to shift during measurement, which can compromise its accuracy [23]. However, a previous study reported better kinematic results using an IMU [10], although the IMU was attached using velcro strips on the top of the foot and the walking speeds were slower in that study, ranging from 1.5 km/h to 5 km/h.

There are several limitations to this study that should be considered when interpreting the data. First, the data was only collected in healthy participants. Consequently, it is questionable whether similar accuracy would be observed in patients with atypical gait patterns. Second, test conditions only included walking and running on a treadmill in standardised laboratory settings, limiting its transferability to overground and real‐world conditions. This approach was chosen to enable continuous measurement over one minute per trial and slope condition while eliminating unnecessary sources of noise. Third, participants wore their own shoes. Indeed, the fundamental differences between the treadmill and the WSS is that insoles measure pressure at the foot‐shoe interface [19]. Thus, the differences in the shape and mechanical properties of the sole may have affected the inter‐individual variability, and thus, the agreement. Still, it is important to validate the WSS regardless of shoe choice and despite possible influence of the shoe as it guarantees the generalisability across different shod conditions.

## CONCLUSION

This study demonstrated that the WSS can generally measure spatiotemporal variables with excellent to good accuracy across various walking speeds and slopes in healthy participants. The findings suggest that the WSS is suitable for gait assessment in a patient's real‐world environment. However, limitations in measuring kinematic variables highlight the need for further refinement, particularly in IMU placement and calibration. Future research should explore its application in populations with atypical gait patterns and real‐world conditions to fully establish its utility and reliability.

## AUTHOR CONTRIBUTIONS

All authors contributed to the study conception and design. Material preparation, data collection and analysis were performed by Jennifer Fayad, Anne Backes, Gaëlle Schurmans, Valeria Serchi and Melanie Eckelt. The first draft of the manuscript was written by Melanie Eckelt and all authors commented on previous versions of the manuscript. All authors read and approved the final manuscript.

## CONFLICT OF INTEREST STATEMENT

The authors declare that Tobias Meyer, Dr. Valeria Serchi and Thomas Solignac are employees of IEE S.A., the manufacturer of the insole investigated in this study. All conflicts of interest have been disclosed in accordance with the journal's guidelines and did not influence the design, execution, analysis, or interpretation of the research. All other authors declare that they have no conflict of interest.

## ETHICS STATEMENT

This study was conducted in accordance with the ethical standards of the Declaration of Helsinki and was approved by the National Ethics Committee for Research (CNER) (Reference: 202212/04 Version 2.0). All participants provided written informed consent prior to participation.

## Supporting information

Figure S1: Bland‐Altman Plots for different gait metrics at 3 km/h (no slope).
Figure S2: Bland‐Altman Plots for different gait metrics at 4.5 km/h (−3° slope).
Figure S3: Bland‐Altman Plots for different gait metrics at 4.5 km/h (−6° slope).
Figure S4: Bland‐Altman Plots for different gait metrics at 4.5 km/h (3° slope).
Figure S5: Bland‐Altman Plots for different gait metrics at 4.5 km/h (6° slope).
Figure S6: Bland‐Altman Plots for different gait metrics at 6 km/h (no slope).
Figure S7: Bland‐Altman Plots for different gait metrics at 9 km/h (no slope).

## Data Availability

The data sets generated during the current study are not publicly available but are available from the corresponding author on reasonable request.
