# Supplementary material for: Validity of a wireless instrumented insole (WalkinSense system) for measuring gait metrics
Source: J Exp Orthop. 2025 Sep 27;12(4):e70438. doi: 10.1002/jeo2.70438 (PMC12475930; doi:10.1002/jeo2.70438)
Supplement: Supplementary file 1 — Figure S1: Bland‐Altman Plots for different gait metrics at 3 km/h (no slope). Figure S2: Bland‐Altman Plots for different gait metrics at 4.5 km/h (−3° slope). Figure S3: Bland‐Altman Plots for different gait metrics at 4.5 km/h (−6° slope). Figure S4: Bland‐Altman Plots for different gait metrics at 4.5 km/h (3° slope). Figure S5: Bland‐Altman Plots for different gait metrics at 4.5 km/h (6° slope). Figure S6: Bland‐Altman Plots for different gait metrics at 6 km/h (no slope). Figure S7: Bland‐Altman Plots for different gait metrics at 9 km/h (no slope). [file JEO2-12-e70438-s001.docx]

Supplementary Documents

Bland-Altman analyses for all gait metrics at different test conditions

| 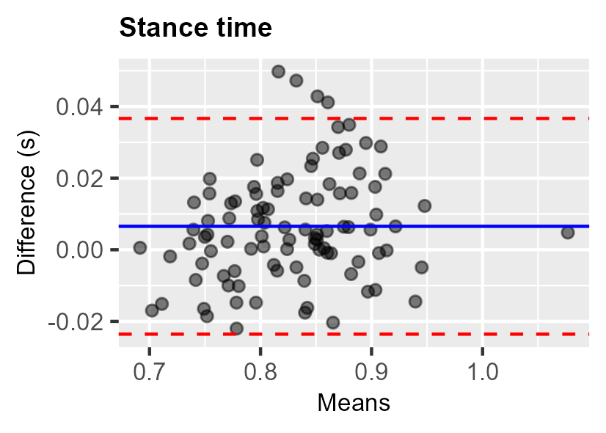 | 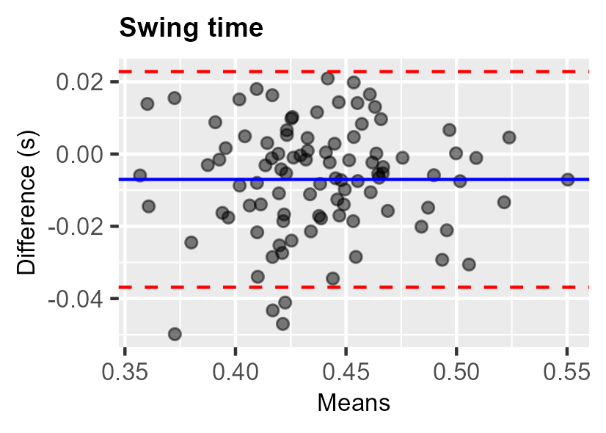 |
| --- | --- |
| 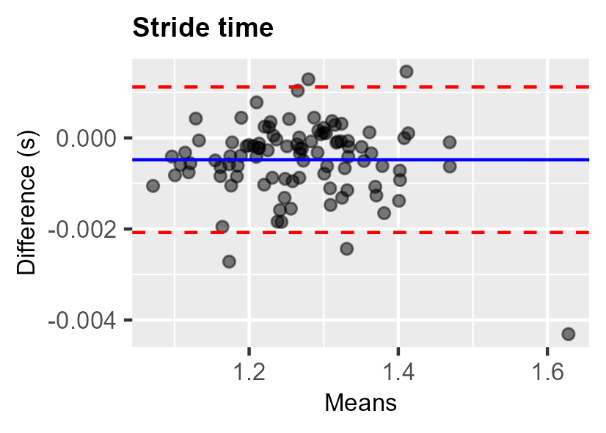 | 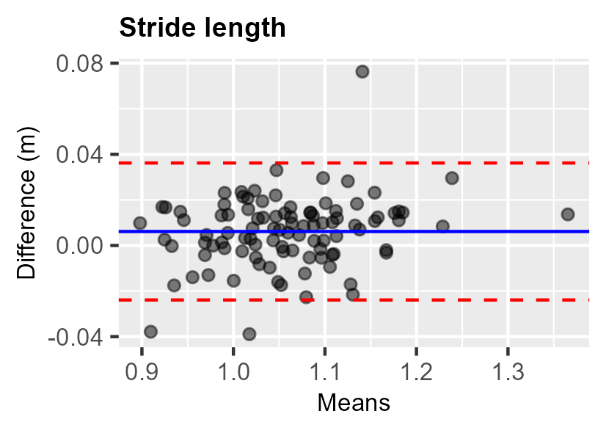 |
| 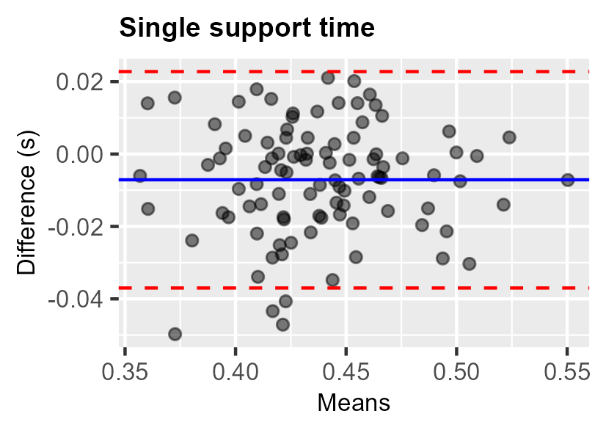 | 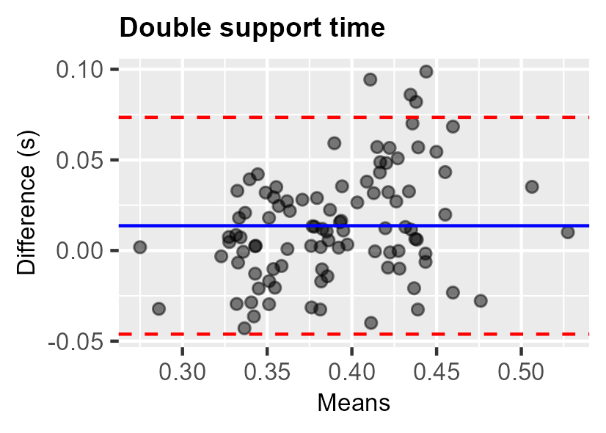 |
| 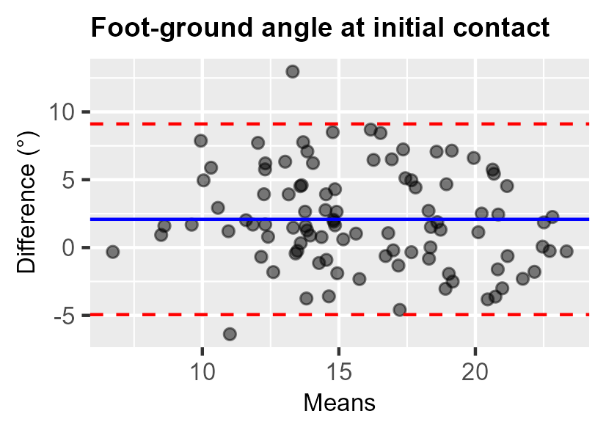 | 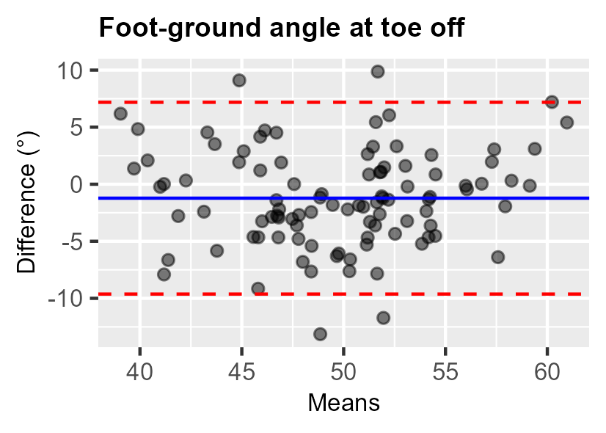 |
|  |  |

Figure S1: Bland-Altman Plots for different gait metrics at 3 km/h (no slope)

| 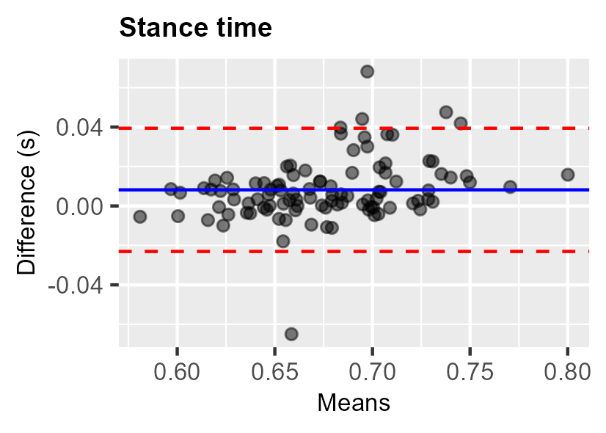 | 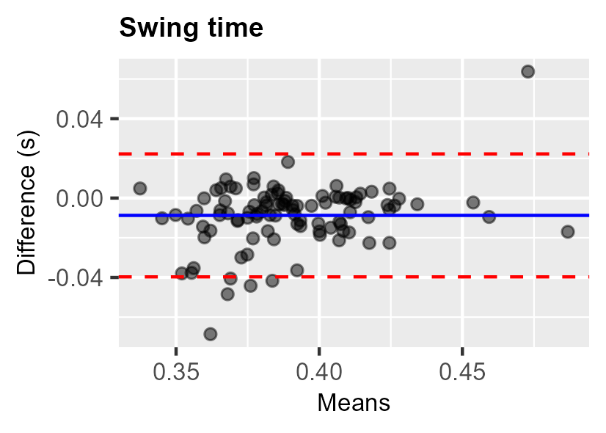 |
| --- | --- |
| 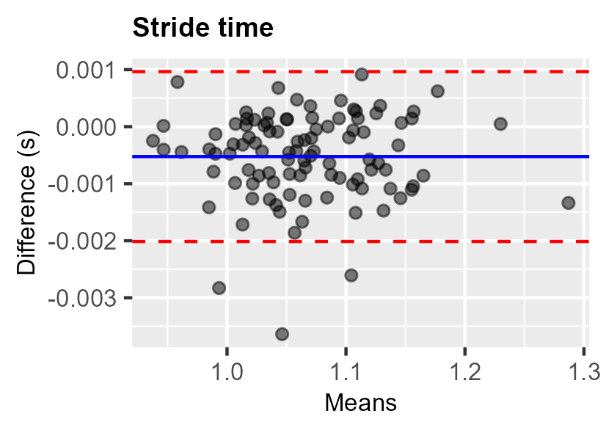 | 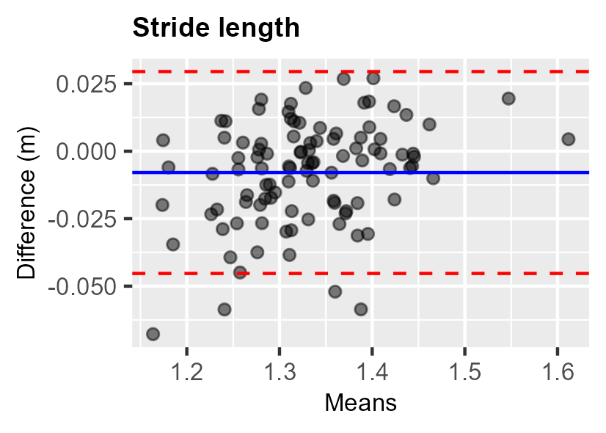 |
| 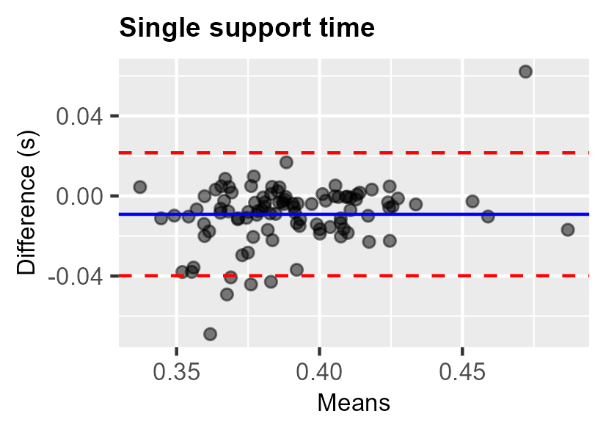 | 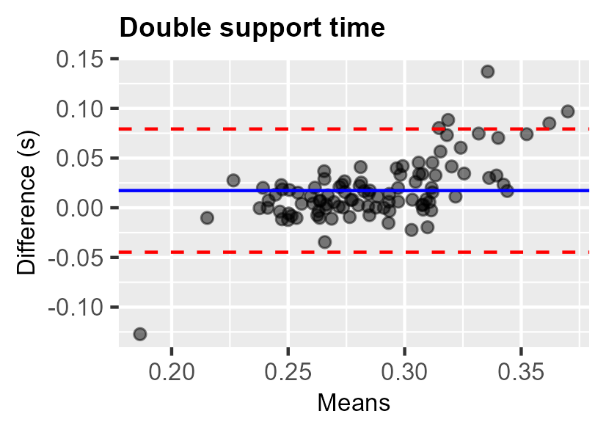 |
| 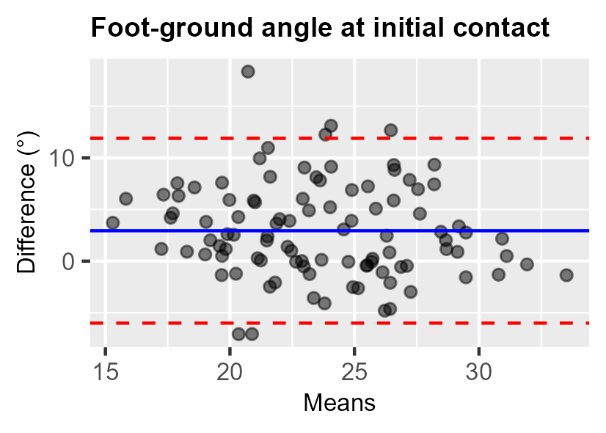 | 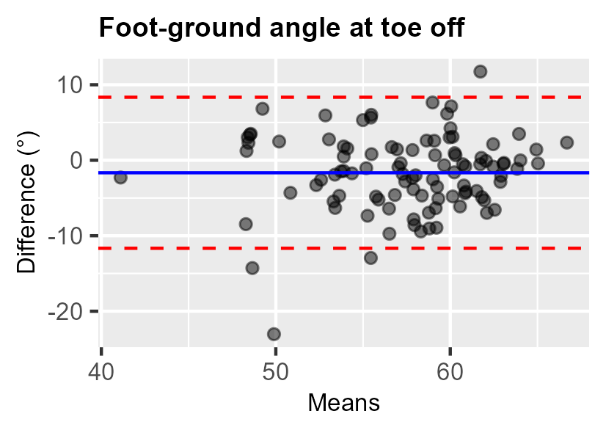 |
|  |  |

Figure S2: Bland-Altman Plots for different gait metrics at 4.5 km/h (-3° slope)

| 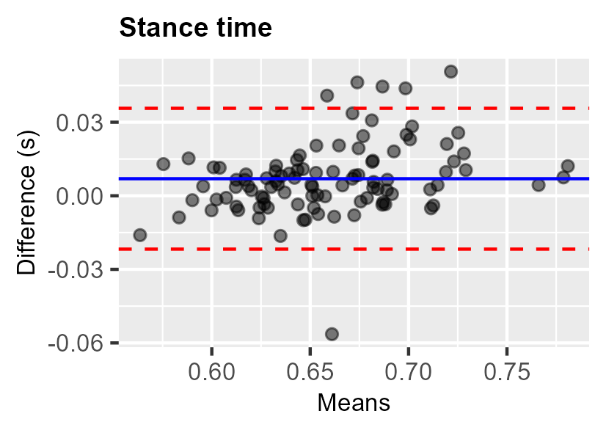 | 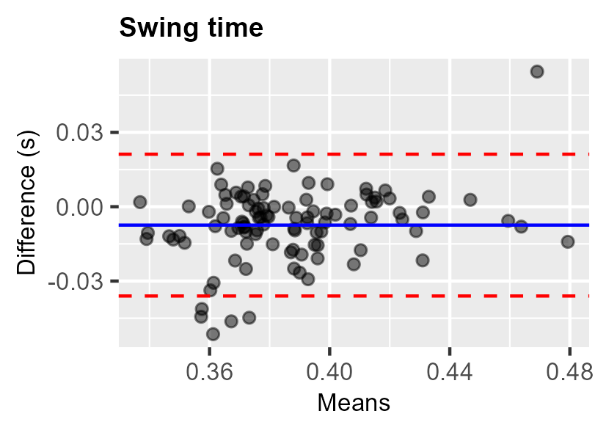 |
| --- | --- |
| 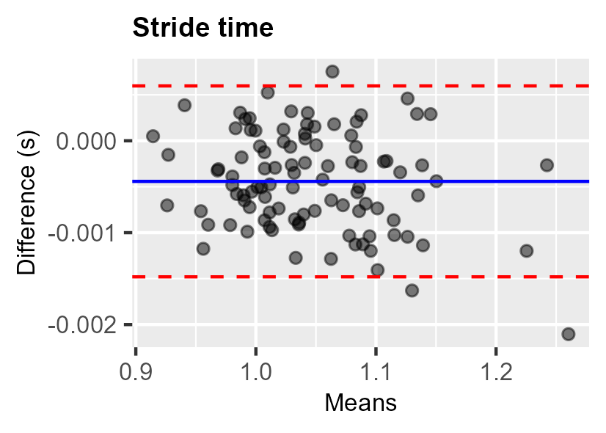 | 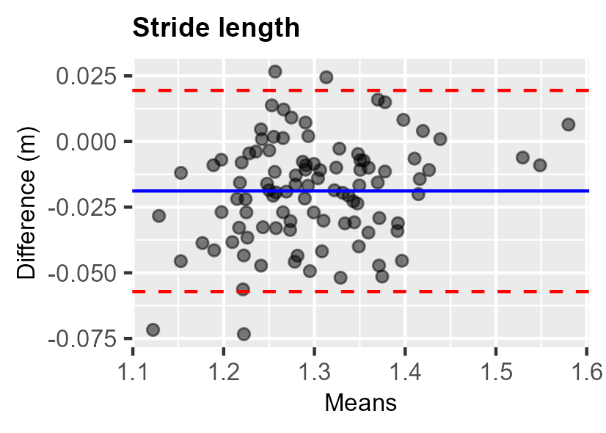 |
| 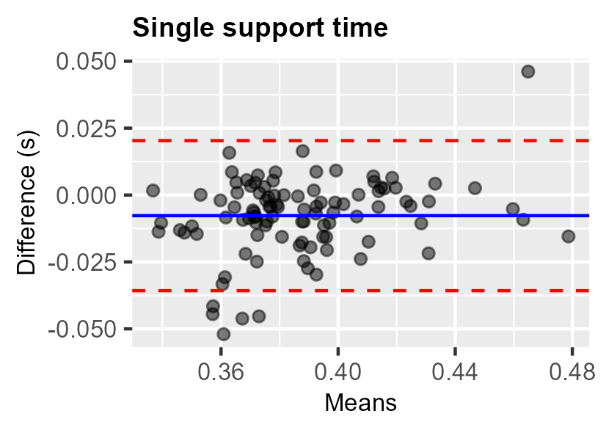 | 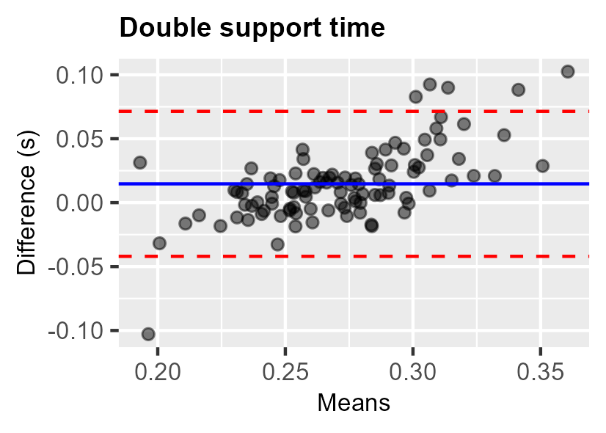 |
| 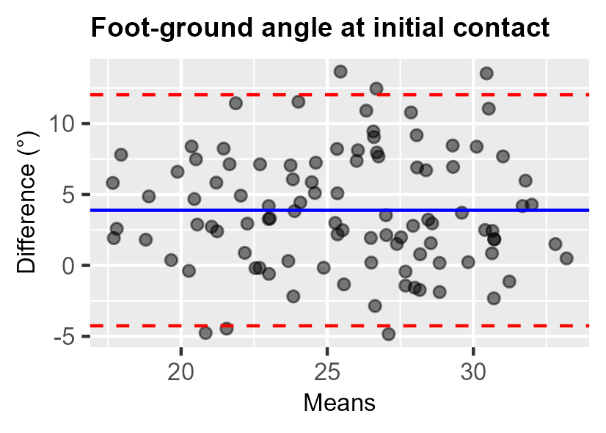 | 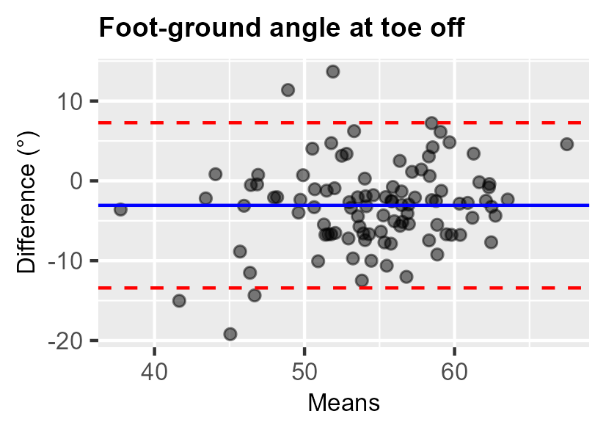 |
|  |  |

Figure S3: Bland-Altman Plots for different gait metrics at 4.5 km/h (-6° slope)

| 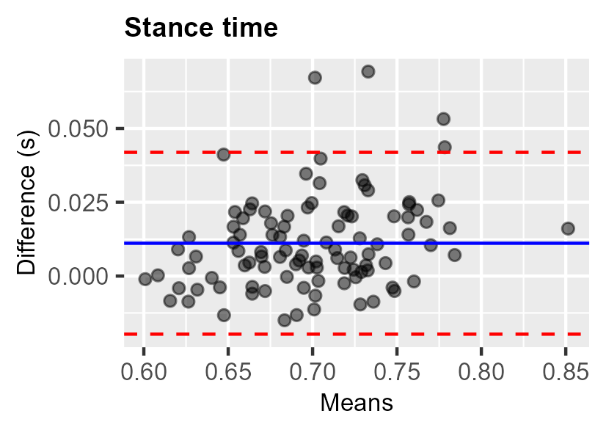 | 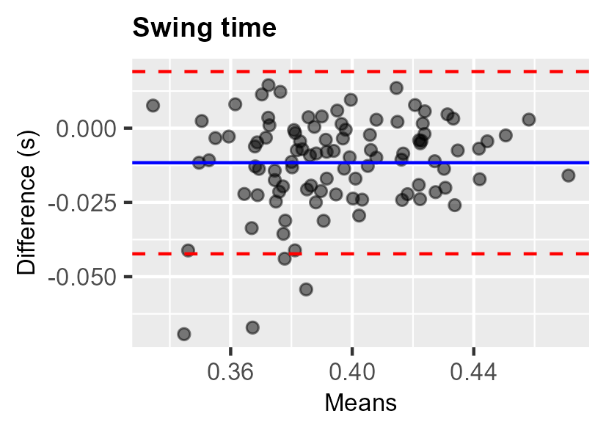 |
| --- | --- |
| 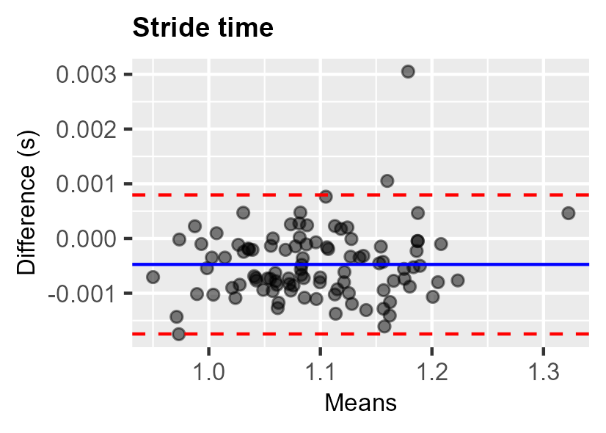 | **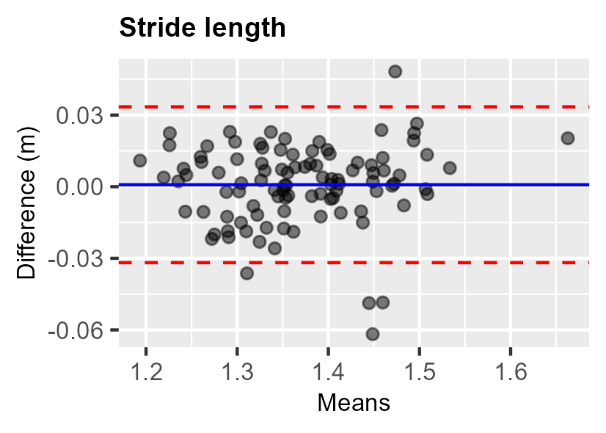** |
| 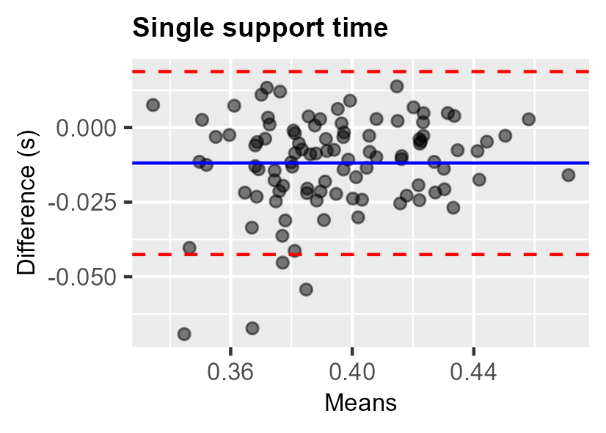 | 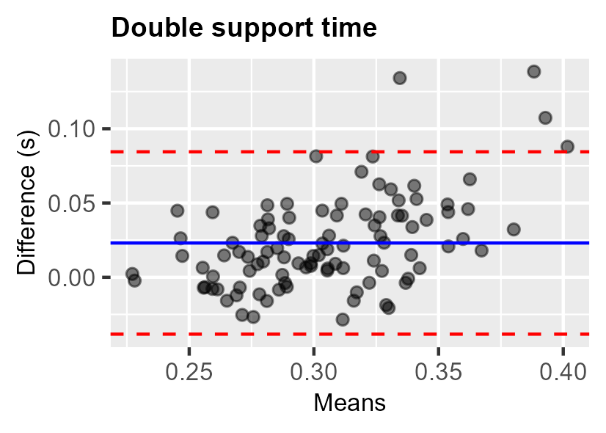 |
| 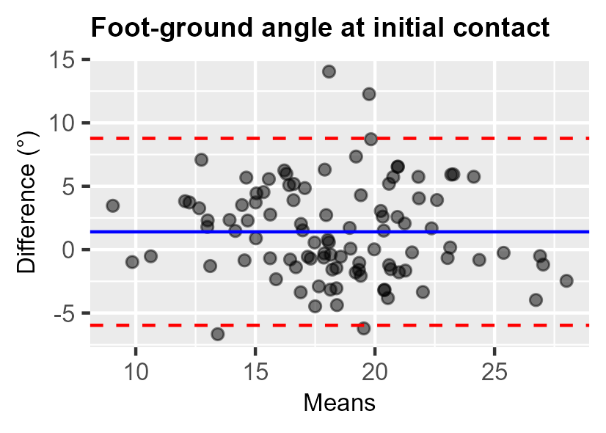 | 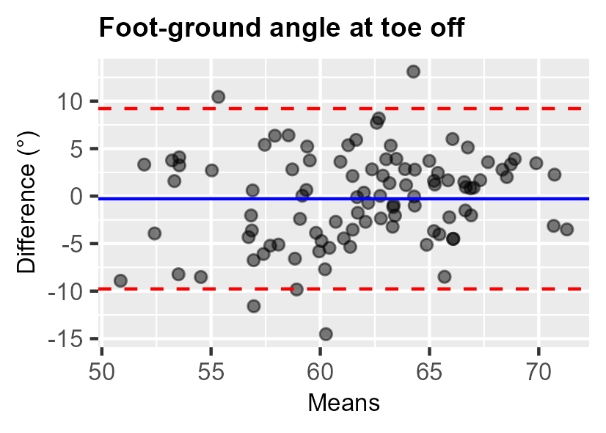 |
|  |  |

Figure S4: Bland-Altman Plots for different gait metrics at 4.5 km/h (3° slope)

| 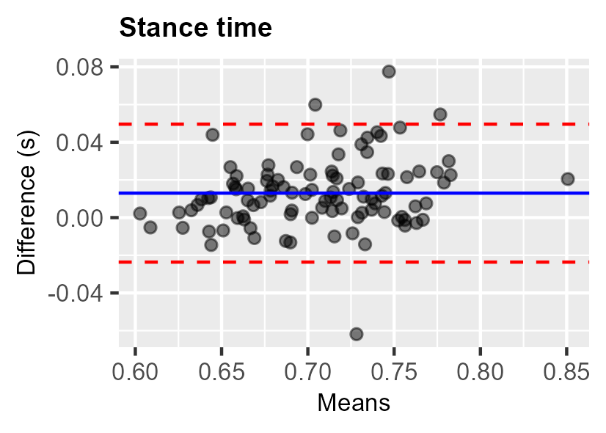 | 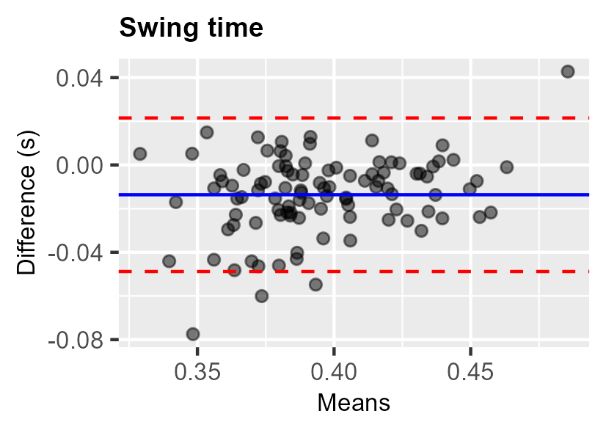 |
| --- | --- |
| 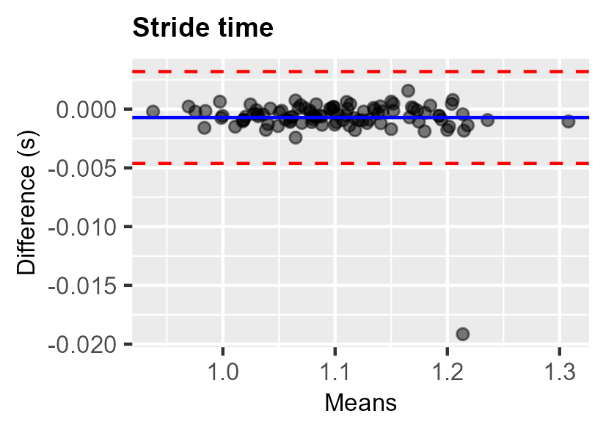 | **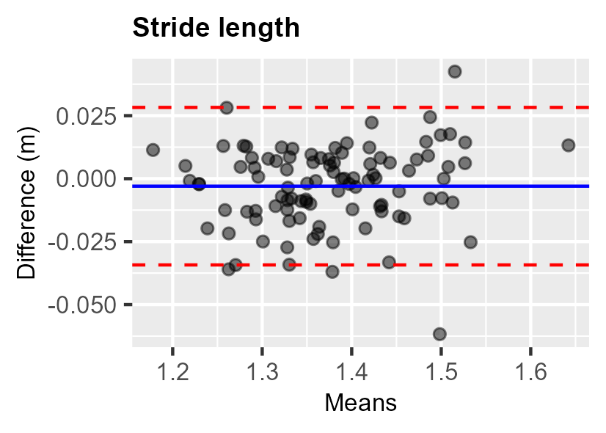** |
| 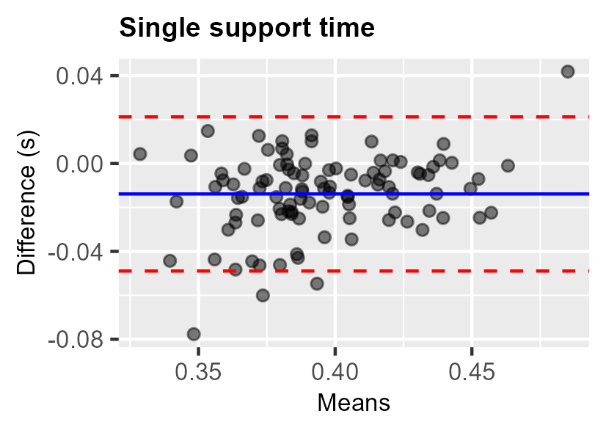 | 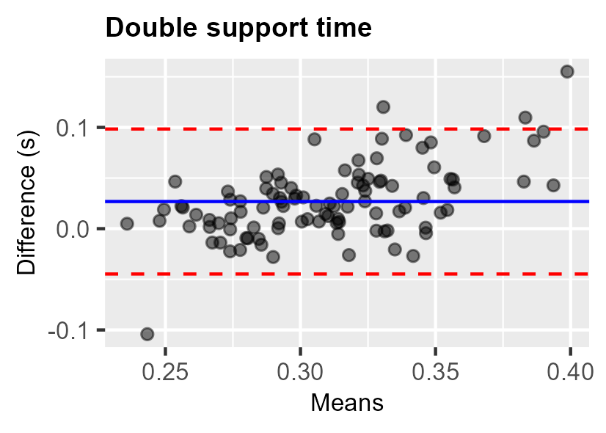 |
| 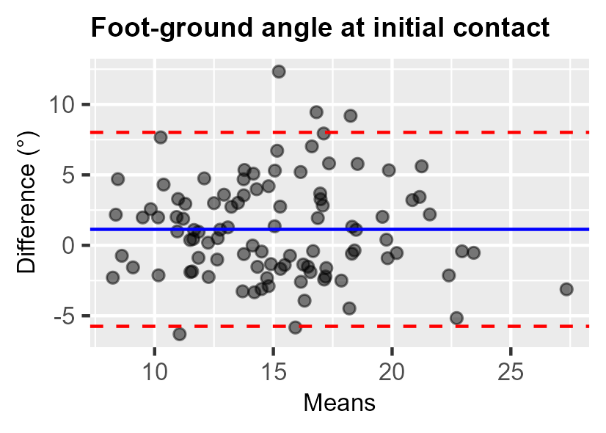 | 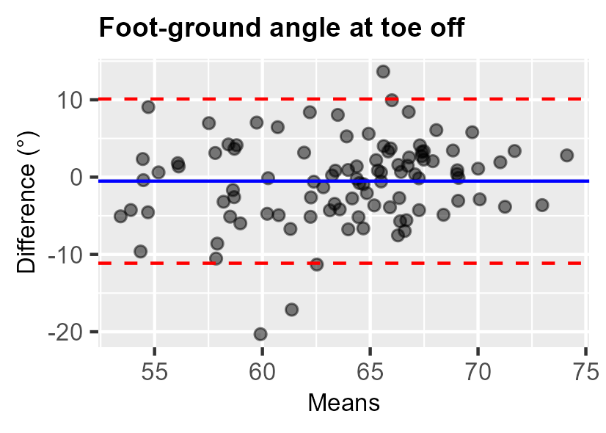 |
|  |  |

Figure S5: Bland-Altman Plots for different gait metrics at 4.5 km/h (6° slope)

| 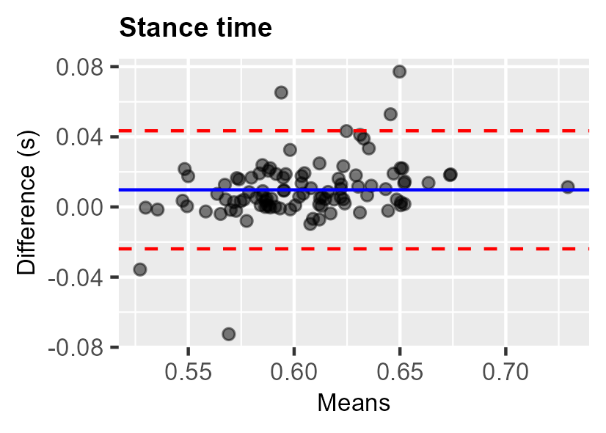 | 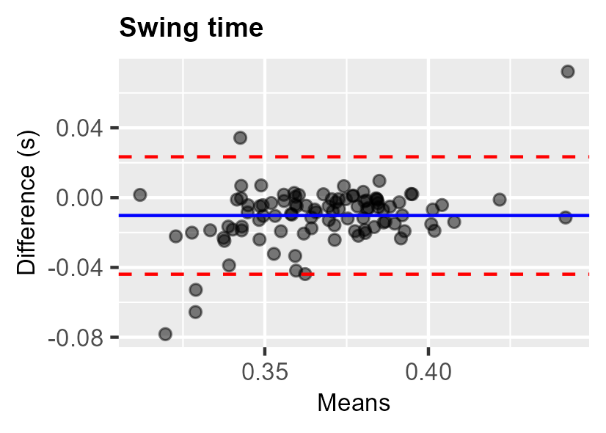 |
| --- | --- |
| 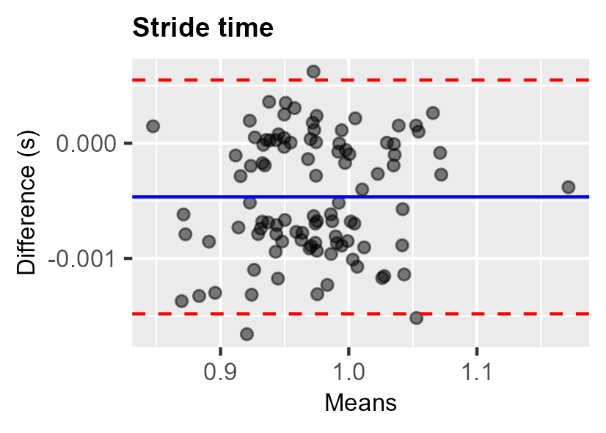 | 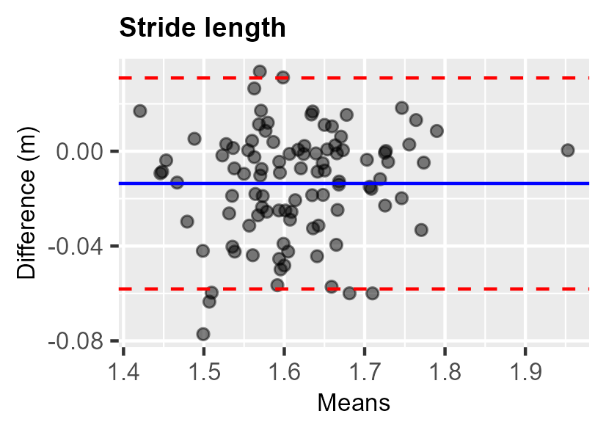 |
| 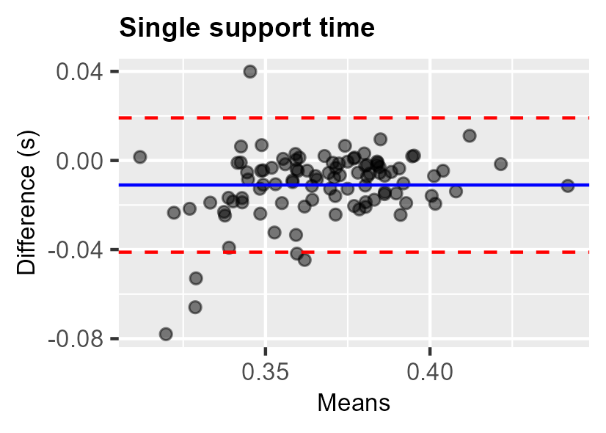 | 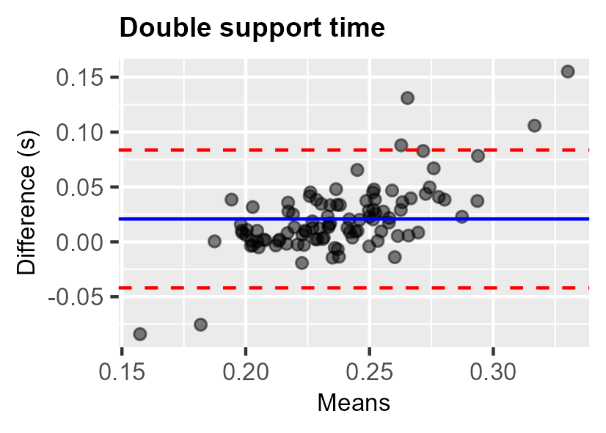 |
| 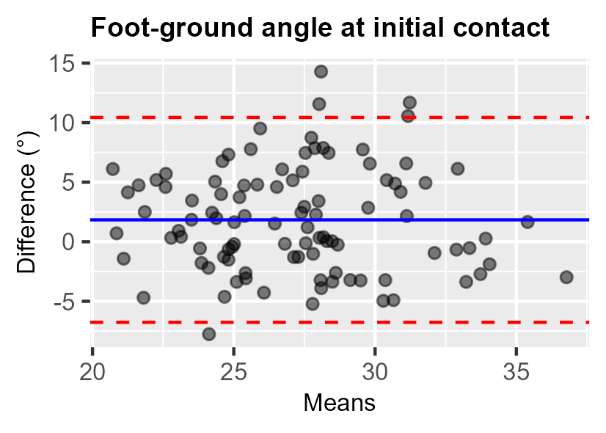 | 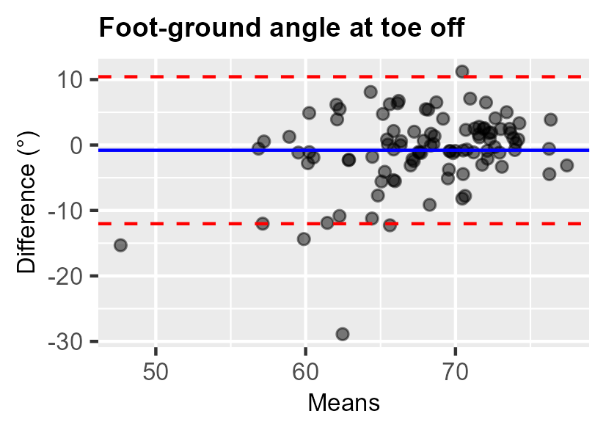 |
|  |  |

Figure S6: Bland-Altman Plots for different gait metrics at 6 km/h (no slope)

| 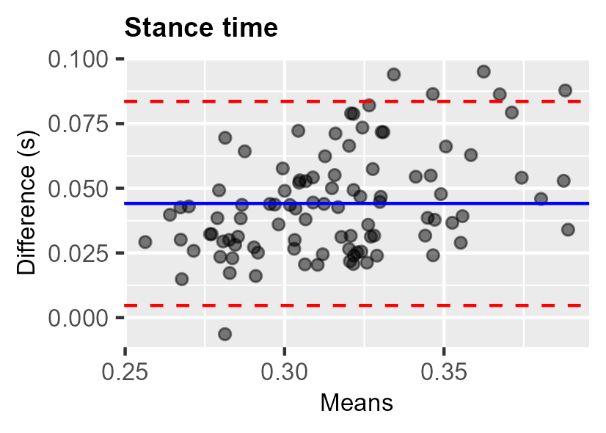 | 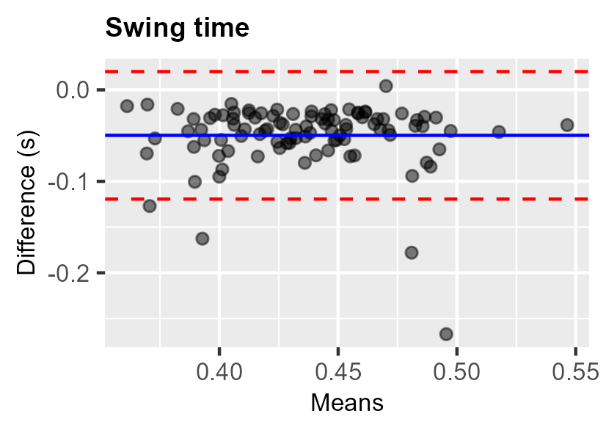 |
| --- | --- |
| 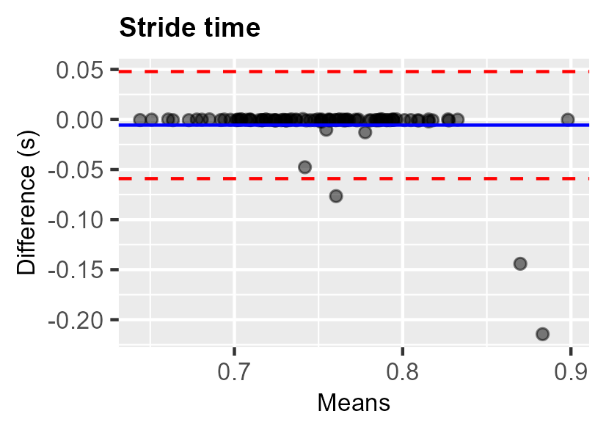 | 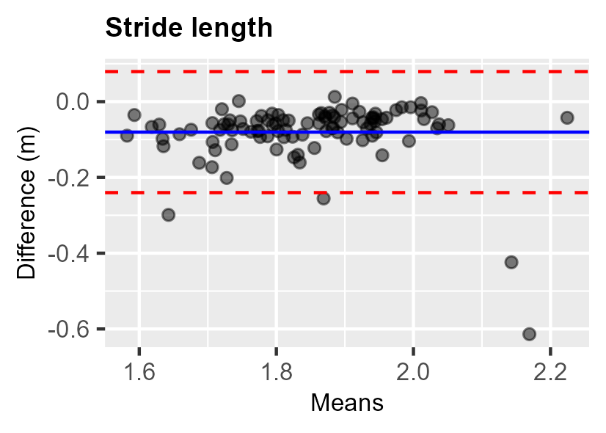 |
| 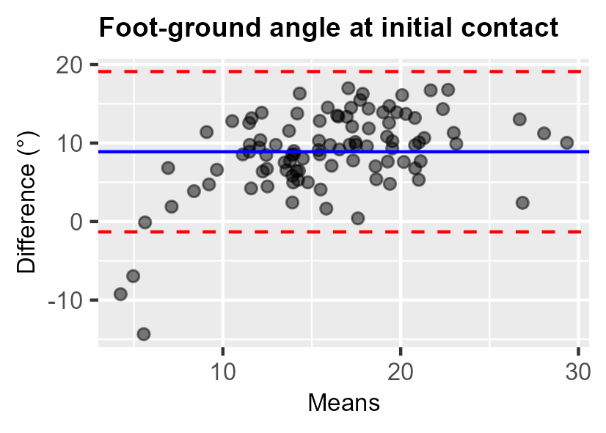 | 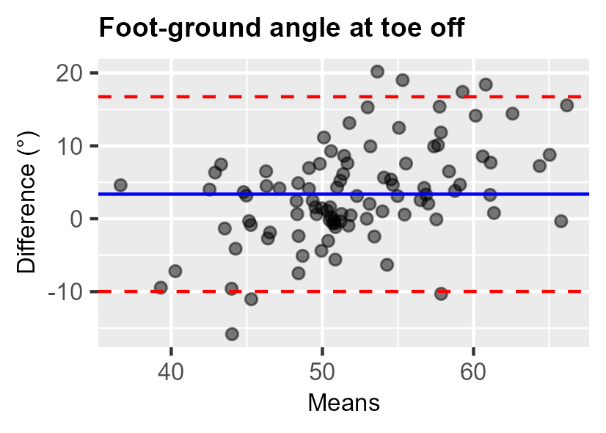 |
|  |  |

Figure S7: Bland-Altman Plots for different gait metrics at 9 km/h (no slope)
